# Radiomics analysis improves ^18^FDG PET/CT-based risk stratification of cytologically indeterminate thyroid nodules

**DOI:** 10.1007/s12020-021-02856-1

**Published:** 2021-09-01

**Authors:** Luca Giovanella, Lisa Milan, Arnoldo Piccardo, Gianluca Bottoni, Marco Cuzzocrea, Gaetano Paone, Luca Ceriani

**Affiliations:** 1 Clinic for Nuclear Medicine and Molecular Imaging, Imaging Institute of Southern Switzerland, Ente Ospedaliero Cantonale, 6500 Bellinzona, Switzerland; 2 Clinic for Nuclear Medicine, University Hospital and University of Zurich, Zurich, Switzerland; 3 Department of Nuclear Medicine, E.O. “Ospedali Galliera”, Genoa, Italy; 4 Faculty of Biomedical Sciences, Università della Svizzera Italiana (USI), Lugano, Switzerland

**Keywords:** ^18^FDG-PET-CT, Indeterminate thyroid nodule, Radiomics, PET metrics, Predictive model

## Abstract

**Purpose:**

As ~25% of cytologically indeterminate thyroid nodules harbour malignancy, diagnostic lobectomy is still performed in many cases. ^18^FDG PET/CT rules out malignancy in visually negative nodules; however, none of the currently available interpretation criteria differentiates malignant from benign ^18^FDG-avid nodules. We evaluated the ability of PET metrics and radiomics features (RFs) to predict final diagnosis of ^18^FDG-avid cytologically indeterminate thyroid nodules.

**Methods:**

Seventy-eight patients were retrospectively included. After volumetric segmentation of each thyroid lesion, 4 PET metrics and 107 RFs were extracted. A logistic regression was performed including thyroid stimulating hormone, PET metrics, and RFs to assess their predictive performance. A linear combination of the resulting parameters generated a radiomics score (RS) that was matched with cytology classes (Bethesda III and IV) and compared with final diagnosis.

**Results:**

Two RFs (shape_Sphericity and glcm_Autocorrelation) differentiated malignant from benign lesions. A predictive model integrating RS and cytology classes effectively stratified the risk of malignancy. The prevalence of thyroid cancer increased from 5 to 37% and 79% in accordance with the number (score 0, 1 or 2, respectively) of positive biomarkers.

**Conclusions:**

Our multiparametric model may be useful for reducing the number of diagnostic lobectomies with advantages in terms of costs and quality of life for patients.

## Introduction

Indeterminate thyroid cytology (i.e., Bethesda class III and IV) corresponds to either benign or malignant lesions at final histology, which are particularly difficult to differentiate by cytology alone. The overall incidence for indeterminate nodules is around 20–25% of all thyroid fine-needle aspiration cytology (FNAC) and the risk of malignancy ranges from 10 to 30% for category III and 25 to 40% for category IV (follicular neoplasm/suspicious for follicular neoplasm). In addition, the use of Bethesda System is heterogeneous across institutions, and there is some degree of subjectivity in the distinction between categories III and IV [1]. Actually, diagnostic lobectomy is still performed in many cases even if most cytologically indeterminate nodules are actually benign at final histopathology examination. Accordingly, a preoperative test or a combination of tests are warranted to prevent unbeneficial diagnostic lobectomies for benign nodules and limit the number of two-stage surgeries for thyroid malignancies, respectively. Advanced preoperative diagnostics are rapidly evolving and include immunocytochemistry, gene mutation analysis, microRNA, sequencing techniques, ultrasonography, elastosonography, computed tomography, sestamibi scintigraphy and [18F]-2-fluoro-2-deoxy-D-glucose positron emission tomography/computed tomography (^18^FDG PET/CT). de Koster et al. extensively reviewed the diagnostic utility of available molecular and imaging biomarkers and found BRAF mutation analysis to be the best rule-in test, while the most accurate rule-out tests were the Afirma^®^ (Veracyte, Austin, USA) gene expression classifier (GEC) and ^18^FDG PET/CT, respectively [2]. Indeed, the excellent performance of ^18^FDG PET/CT in excluding malignancy relies on visual assessment of nodules without discernible uptake being scored as negative (i.e., benign) and nodules with any discernible uptake marked as suspicious (i.e., malignant). In order to improve the diagnostic accuracy among visually 18FDG-avid nodules, semiquantitative analysis of ^18^FDG uptake using the maximum standardized uptake value (SUVmax) was adopted [3]. The SUVmax is generally significantly higher in malignant than in benign lesions but, unfortunately, no threshold can accurately differentiate benign from malignant nodules [4]. Recently, Ceriani et al. proved that the addition of radiomics features (RFs) to conventional PET metrics improves identification of ^18^FDG-avid thyroid incidentalomas (TIs) at high risk of malignancy. In particular, a model based on total lesion glycolysis (TLG), SUVmax and shape_Sphericity allows prediction of a final diagnosis, providing useful information for the management of TIs [5]. The present study was undertaken to evaluate the ability of PET metrics and RFs to predict the final diagnosis of cytologically indeterminate and ^18^FDG-avid thyroid nodules.

## Materials and methods

### Study design and patient selection

We retrospectively enrolled 85 patients with thyroid nodules who underwent PET/CT scans between February 2013 and November 2019. Seventy-five cases were included from the database of the Nuclear Medicine Department of the E.O. Ospedali Galliera (Genoa, Italy) and ten from the Clinic of Nuclear Medicine and PET-CT centre of the Imaging Institute of Southern Switzerland (Bellinzona, Switzerland). The study inclusion criteria were as follows: (i) an ^18^FDG-avid thyroid nodule with (ii) indeterminate cytological report (Bethesda III and IV) and (iii) a final histological diagnosis. After the preliminary enrollment, seven patients with a lesion <3 mL were excluded to avoid undersampling and to minimize the statistical fluctuations related to small volumes. Therefore, 78 patients were included in the final analysis. The PET/CT imaging was obtained within 1 month before surgery in all cases.

### PET/CT image analysis and radiomics features extraction

^18^FDG PET/CT images were acquired with integrated PET/CT scanners (Genoa: Discovery ST, GE Medical Systems, United States; Bellinzona: Biograph mCT40 scanner, Siemens, Germany) applying comparable standard protocols in accordance with the European Association of Nuclear Medicine guidelines [3]. Acquisitions started 60 ± 5 min following the i.v. injection of ^18^FDG (2.5–3 MBq/Kg) to the patients fasting for at least 6 h. PET images (CT corrected for attenuation) were reconstructed with standard iterative 3D algorithms including Time-of-Flight and Point-Spread Function corrections.

### ^18^FDG PET/CT metrics and radiomics features

An expert nuclear medicine physician (L.C.) centrally analyzed all the reconstructed ^18^FDG PET/CT images with dedicated software (MM Oncology, Syngo.via; Siemens) following a standardized protocol already applied and published [5]. Briefly, all thyroid focal PET findings were countered with a three-dimensional fixed threshold algorithm setting the mean SUV of the contralateral lobe as the threshold to identify the borders between pathological and normal tissue [5]. This method, already applied in a previous study [5], is arbitrarily based on estimating the SUVmean of the contralateral lobe to characterize the FDG uptake of the normal tissue surrounding the thyroid nodules. Different from the most used criteria (i.e., percentage cut-offs, such as 40% or 50% of the lesion SUVmax), this threshold allows to better identify the borders between pathological and normal tissue independently of thyroid nodule uptake/characteristics. Metabolic tumor volume (MTV) and maximum and mean SUVs (SUVmax and SUVmean, respectively) of the lesion were measured automatically. The TLG was calculated as the product of the SUVmean and MTV [6]. RFs were then extracted from each segmented volume using PyRadiomics software package version 2.2.0. To standardize the extraction process, gray-level intensities and voxel dimensions of the original images were preliminarily resampled following the Image Biomarkers Standardization Initiative recommendations [7]. From the segmented volumes, 107 standardized features evaluating different metabolic characteristics of the lesion were initially extracted. They included 14 shape-based features, 18 first-order statistics features, and 75 matrix-based features (24 gray-level co-occurrence matrix [GLCM], 16 gray-level run length matrix, 16 gray-level size zone matrix, 5 neighboring gray tone difference matrix, and 14 gray-level dependence matrix based). Meaning and mathematical descriptions of these RFs are reported in detail in the PyRadiomics documentation. The complete list of features and extraction settings details are reported in Supplementary Information.

### Radiomics features analysis

Because the images were acquired from two different centres, each RF was first standardized using the *Z*-score (with *Z* = [(µ-μ ®)/ σ], where µ, μ ®, and σ are the value, the mean and the standard deviation, respectively) [8].

Taking into account the RF redundancy, we used Spearman’s correlation test (*p* < 0.05) with false discovery rate correction to reduce the overfitting problem of discarding features highly correlated to the SUVmax and MTV (correlation coefficient > 0.7) [9]. We used the least absolute shrinkage and selection operator (LASSO) logistic regression (with tenfold cross validation) to select the most informative parameters of malignancy [10].

Serum TSH levels in the upper-normal range were recently associated with an increased risk of thyroid malignancy in patients affected by thyroid nodules with indeterminate cytology and proposed as an easily performed additional tool for decision-making in patients with indeterminate cytological findings [11]. Accordingly we added TSH levels, together TLG to RFs (first-order and shape PyRadiomics parameters comprised SUVmax, SUVmean and MTV) in our analysis.

### Statistics

Continuous variables were expressed as median and interquartile range and their distribution between groups was compared using the Mann–Whitney U-test. Differences between the frequencies of categorical data were assessed with the chi-square test or the Fisher exact test, as applicable. The ability of the radiomics score (RS) to discriminate subgroups with different histology was assessed by using the receiver operator characteristic area under the curve (ROC-AUC) analysis, and Youden’s coefficient method was used to estimate the optimal cut-off point for discriminating malignant from benign non-Hürthle cell nodules. The model was then retested on the whole patient population. The performance of cytology with respect to RS was evaluated and a logistic stepwise regression function was used for multivariate analysis including both parameters. To evaluate the robustness and generalizability of our model, considering the sample size of the study, a 1000-resampled bootstrapping was performed as a cross-validation procedure. A *p* value < 0.05 was considered statistically significant. Statistical analyses were conducted using the RStudio 1.2.1335 software package (RStudio, PBC; Boston, MA) with R version 4.0.355 and MedCalc^®^ Statistical Software version 15.8, as appropriate.

## Results

### Patient characteristics

The main clinical characteristics of the studied cohort are summarized in Table 1. None of enrolled patients was taking thyroid hormones or antithyroid drugs.

**Table 1:** Patient flow and results of diagnostic work-up

Characteristic	Total (*n* = 78)	Benign lesions (*n* = 55)	Malignant lesions (*n* = 23)	*P* value
Gender, F/M	58/20 (64%/36%)	43/12 (78%/22%)	15/8 (65%/35%)	0.13
Age, years, median (range)	59 (54–74)	57 (48–68)	63 (52–79)	0.06
TSH, mUI/L, median (range)	1.7 (1.1–2.3)	1.7 (1.1–2.3)	1.8 (1.0–2.3)	0.82
Largest diameter, mm, median (range)	24 (18–34)	24 (20–33)	23 (18–44)	0.76
Bethesda class III/IV	35/43 (45%/55%)	30/25 (54%/46%)	5/18 (22%/78%)	0.008
Final diagnosis				
	Follicular adenoma	42	–	
	Hürthle cell adenoma	13	–	
	PTC	–	11	
	FTC	–	10	
	PTC-FV	–	2	

*F* female, *FTC* follicular thyroid carcinoma, *M* male, *PTC-FV* follicular variant of papillary thyroid carcinoma, *TSH* thyroid stimulating hormone

Seventy-eight patients (58 female and 20 male; median age, 59 years) were selected for the present analysis. Lesions had a median size of 24 mm (18–34 mm) and the median TSH value was 1.7 mIU/L (1.1–2.3 mIU/L). According to the Bethesda system, 35 and 43 patients had cytology classified as class III and IV, respectively. As summarized in Table 1, 23 of 78 (29%) nodules were malignant (11 papillary, 10 follicular and 2 mixed carcinomas) and 55 (71%) were benign (42 hyperplastic nodules and 13 Hürthle cell adenomas). In particular, 5 of 35 (14%) Bethesda III and 18 of 43 (42%) Bethesda IV nodules were histologically malignant (*χ*2 test, *p* = 0.0083). The cytological results predicted the risk of malignancy with a global accuracy of 62% and negative (NPV) and positive (PPV) predictive values of 86% and 42%, respectively.

### PET metrics and radiomics analysis

Among the 107 RFs extracted, 65 showed to be highly correlated to SUVmax and/or MTV in the testing set and therefore were removed from the subsequent analysis (detailed list in Supplementary Table 2). The LASSO logistic regression (including the remaining 42 RFs, TLG and TSH) identified two non-redundant predictors of malignancy, i.e., shape_Sphericity and glcm_Autocorrelation with regression coefficients equal to −0.157 and −0.285, respectively (Table 2). The shape_Sphericity describes the roundness of the lesion compared to a sphere. The glcm_Autocorrelation measures the magnitude of the fineness and coarseness of the uptake texture, with a higher value indicating a texture with more pairs with high gray levels [7]. The continuous RS obtained as a linear combination of the two RFs weighted according to their respective LASSO coefficients, showed a significant ability to predict malignancy (AUC = 0.733, 95% CI, 0.609–0.835).

**Table 2:** Selected features from LASSO logistic regression analysis

Feature	Regression coefficient
shape_Sphericity	−0.157
glcm_Autocorrelation	−0.285

The shape_Sphericity is an index of the roundness of the shape of the lesion, with respect to a sphere. The glcm_Autocorrelation is a measure of the magnitude of the fineness and coarseness of the uptake texture

### Multiparametric predictive model

Based on the above results, we tested the predictive power of the dichotomized RS composed of the two selected features in patients with non-Hürthle cell lesions and the whole patient population, respectively.

### Non-Hürthle cell lesions

**Fig. 1:**
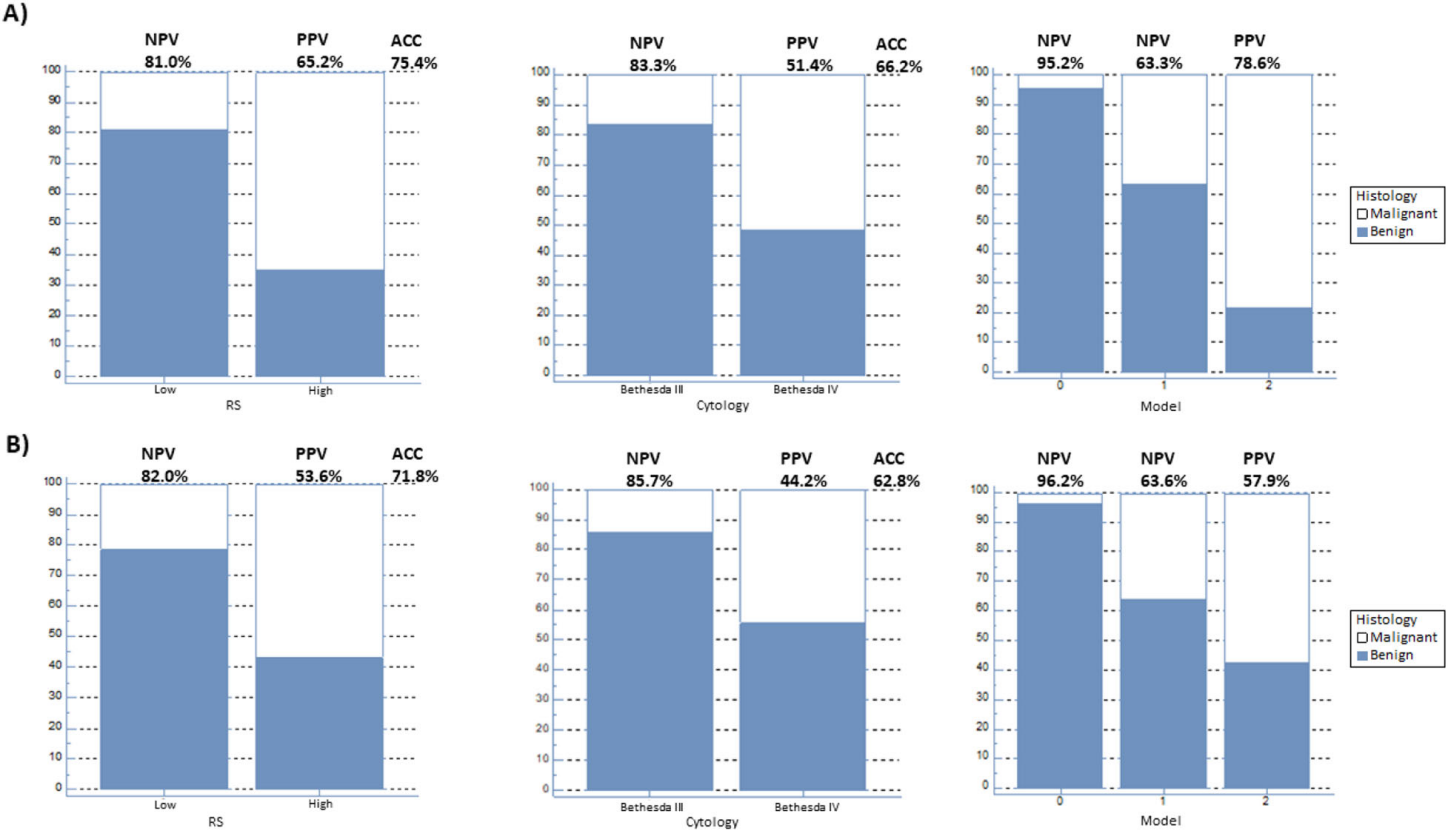
Performance of RS, cytology and the predictive model for (**A**) non-Hürthle and (**B**) Hürthle and non-Hürthle lesions. The predictive model includes the three subgroups characterized by an increasing number (score 0–2) of the two parameters. ACC accuracy, NPV negative predictive value, PPV positive predictive value, RS radiomics score

After the exclusion of 13 patients carrying Hürthle cell adenomas, the analysis was first performed in the remaining 65 patients. In this population the ROC analysis of the RS estimated an optimal cut-off point of 0.049 (AUC = 0.730, *p* = 0.0007) for identifying patients with thyroid cancer, with a sensitivity, specificity and accuracy of 65% (95% CI, 43–84%), 81% (95% CI, 66–91%) and 75%, respectively (Fig. 1a). The cytological results (Bethesda III and IV) predicted the final diagnosis of thyroid cancer with a sensitivity, specificity and accuracy of 78%, 60% and 66% (*χ*2 test, *p* = 0.0037), respectively. The multivariate analysis was performed by a logistic regression function including dichotomized RS and cytological results. Increased RS (odds ratio [OR], 9.5; 95% CI, 2.6–34.9; *p* = 0.0007) and Bethesda class IV (OR, 6.5; 95% CI, 1.7–25.3; *p* = 0.007) remained independently associated with higher risk of malignancy (AUC = 0.818, 95% CI, 0.703–0.903). The 1000-resampled bootstrapping method validated the multivariate analysis results with an optimism-corrected AUC of 0.728 (*p* < 0.0001). A predictive model based on the combination of these two parameters was then built and tested. In this model, three subgroups of lesions were defined by a score of 0, 1 or 2, representing the number of positive biomarkers (Table 3). This model was effective in stratifying the risk of malignancy (*χ*^2^, *p* < 0.0001). In more detail, prevalence of thyroid cancer increased from 5% (1/21) to 37% (11/30) and 79% (11/14) for patients with scores of 0, 1 and 2, respectively (*χ*^2^ trend, *p* < 0.0001). Compared with the a priori risk of malignancy in the whole population, the risk of malignancy was similar in the group with a score of 1 (*χ*^2^ test, *p* = 0.697) but significantly decreased in the group with a score of 0 (*χ*^2^ test, *P* = 0.017) and significantly increased in the group with a score of 2 (*χ*^2^ test, *p* = 0.0007), respectively. In summary, a score of 0 identified benign nodules with a 95% NPV, while a score of 2 predicted malignancy with a 79% PPV. Compared with either RS or cytological results considered separately, this model consistently increased the PPV and showed slight improvement of the NPV (Fig. 1).

**Table 3:** Logistic regression results for non-Hürthle lesions and all lesions, respectively

Non-Hürthle lesions							
Variable	OR (95% CI)	*p* value	Sensitivity (%)	Specificity (%)	Accuracy (%)	NPV (%)	PPV (%)
*Univariate analysis*							
RS	7.97 (2.52–25.2)	0.0002	65.2	81	75.4	81	65.2
Cytology^a^	5.3 (1.6–17.0)	0.0028	78.3	59.5	66.2	83.3	51.4
*Multivariate analysis*							
RS	9.5 (2.6–34.9)	0.0007					
Cytology^a^	6.5 (1.7–25.3)	0.007					
*Model*							
Overall		<0.0001					
Score 0	1					95.2	
Score 1	11.6 (1.4–98.5)					63.3	
Score 2	73.3 (6.8–792.2)						78.6
*All lesions*							
*Univariate analysis*							
RS	5.3 (1.9–14.8)	0.0012	62.5	75.9	71.8	82	53.6
Cytology^a^	4.8 (1.5–14.6)	0.0034	79.2	55.6	62.8	85.7	44.2
*Multivariate analysis*							
RS	4.6 (1.6–13.7)	0.0054					
Cytology^a^	4.1 (1.3–13.4)	0.0183					
*Model*							
Overall		<0.0001					
Score 0	1					96.2	
Score 1	14.3 (1.7–119.1)					63.6	
Score 2	34.4 (3.8–309.2)						57.9

*NPV* negative predictive value, *OR* odds ratio, *PPV* positive predictive value, *RS* radiomics score

^a^Cytology data cover lesions identified as Bethesda class IV

### All lesions

Considering the whole population (78 patients, including 13 patients carrying Hürthle cell adenomas), the dichotomized RS showed an accuracy of 72% in predicting the risk of malignancy with a sensitivity and a specificity of 63% and 76%, respectively (*χ*^2^ test, *p* = 0.0012). The multivariate analysis confirmed increased RS (OR, 4.6; 95% CI, 1.6–13.7; *p* = 0.0054) and Bethesda class IV (OR, 4.1; 95% CI, 1.3–13.4; *p* = 0.0183) as independent predictors of a final diagnosis of thyroid cancer. Therefore, the final model combining the two parameters showed good discriminating performance (*χ*^2^, *p* = 0.0004) (Table 3). In particular, the prevalence of malignant nodules increased from 4% (1/26) to 36% (12/33) and 58% (11/19) for patients with scores of 0, 1, and 2, respectively (*χ*^2^ trend, *p* = 0.0001). Overall, a score of 0 confirmed a high NPV (96%), while the PPV for a score of 2 was reduced (58%) following inclusion of Hürthle cell lesions (Fig. 1).

## Discussion

Assessment of cytologically indeterminate nodules is still a critical challenge for thyroidologists who need to balance the risk of cancer misdiagnosis and that of overtreatment with associated (potential) side effects and costs.

Molecular imaging using 18FDG provides useful data on biological behavior and aggressiveness of thyroid nodules [12]. In particular, available data consistently prove high diagnostic performance of visually interpreted 18FDG PET/CT in cytologically indeterminate nodules with pooled sensitivity and NPV values of 95% and 96%, respectively [13]. Indeed, De Geus-Oei et al. and Sebastianes et al. argued that preoperative ^18^FDG PET/CT could reduce the number of futile hemithyroidectomies by 66% and 39%, respectively [14, 15].

More recently, the 5-year cost-effectiveness of routine ^18^FDG PET/CT in patients with cytologically indeterminate thyroid nodules was also assessed. Routine ^18^FDG PET/CT could prevent 47% of inappropriate diagnostic lobectomies, reducing costs and increasing patients’ quality of life. Moreover, ^18^FDG PET/CT compared favorably with a GEC test and molecular marker panel [16]. All considered, a visually ^18^FDG-negative nodule carries a very low risk of malignancy but the accuracy of ^18^FDG PET/CT is limited in ^18^FDG-avid nodules [17].

A different approach to ^18^FDG PET/CT evaluation has been recently proposed based on the assessment of ^18^FDG distribution within thyroid nodules. Preliminary data suggested that metabolic heterogeneity might differentiate benign from malignant nodules more accurately than conventional PET metrics [18]. Sollini et al. reported interesting results by evaluating histogram-based and matrix-based features with textural analysis [19, 20]. In a previous study [5], Ceriani et al. demonstrated good accuracy of a model incorporating PET metrics and RF in distinguishing benign from malignant TIs. Briefly, shape_Sphericity was the best predictor, classifying 82% of TIs correctly. Moreover, TLG, SUVmax, and shape_Sphericity retained statistical significance in a multivariate analysis, and malignancy rate increased from 7 to 100% in accordance with the number of positive parameters present. To the best of our knowledge, our study is the first attempt to integrate textural features and PET metrics in a multiparametric predictive model for cytologically indeterminate thyroid nodules. In such a clinical challenging population, we identified a radiomic signature including shape_Sphericity and glcm_Autocorrelation as an effective tool for discriminating benign from malignant lesions.

In fact, the analysis showed that spherical lesions with rough texture had lower risk of malignancy. We can interpret this result considering that the loss of sphericity is a consequence of the abnormal/asymmetrical growth of the tumor lesions and different values of glcm-Autocorrelation can discriminate between the fine structure of tissue with high cell density that characterizes the micro-follicular lesions (more frequently malignant) and a more coarse structure of macro follicular nodules (more frequently benign). Moreover, we demonstrated that a predictive model combining RS and Bethesda classes accurately stratified the risk of malignancy of cytologically indeterminate and ^18^FDG-avid thyroid nodules.

Notably, the prevalence of malignancy may significantly differ in class III and IV nodules, depending by the prevalence of malignancies in the local population and expertize of cytopathologist and in our series the prevalence of malignancy was consistently higher in class IV than class III. Notwithstanding, as the main result of our study, radiomic signatures retained independency in multivariate analysis and the addition of PET/CT radiomics in a multiparameter model refined the diagnostic accuracy in both categories.

Separate analyses were performed with and without Hürthle cell lesions as high ^18^FDG uptake is observed in both benign and malignant Hürthle cell nodules, and higher SUVmax values are generally recorded in Hürthle cell adenoma than in other benign nodules [21]. Interestingly, our model retained a good discriminating performance even when Hürthle cell lesions were included (PPV 79% and 58% without and with Hürthle cell lesions, respectively). Notably, a score of 0 excluded malignancy with 96% NPV even in Hürthle cell lesions, which represents a significant improvement compared with visual assessment of PET/CT with or without SUVmax incorporation. Compared with a previous patient population in whom ^18^FDG-avid TIs were incidentally detected during PET/CT [5], some differences were found in the current analysis. First, standard PET metrics features describing glucose metabolic rate were not able to discriminate benign from malignant lesions. Second, the lesion shape (i.e., sphericity) remained relevant when combined with a texture feature describing the intra-lesion heterogeneity. Such differences are likely due to subtle metabolic differences in benign and malignant follicular-patterned lesions, making conventional parameters, such as SUVmax, less relevant. Malignant lesions are histologically characterized by capsule and vascular invasion and apoptosis/necrosis potentially resulting in shape distortion and inhomogeneous tissue structure, making the role of textural features preeminent in differentiating benign from malignant follicular-patterned lesions.

Our retrospective, multicentre study has some limitations. First, a validated threshold value to segment 18FDG-avid thyroid nodules has not yet been defined. However, our approach was based on arbitrary selection of the SUVmean of the contralateral lobe to define the actual nodules’ volume independently of their metabolic activity and thus increase accuracy and reproducibility of radiomics analysis.

Second, we analyzed only lesions larger than 10 mm; thus, our findings may not be applicable to smaller nodules. On the other hand, current recommendation is to not perform FNAC in nodules, including ^18^FDG-avid nodules, less than 10 mm in largest diameter [22].

Third, while up to 25% of all thyroid nodules FNAC result in indeterminate results, a relatively small series of patients was enrolled in our study. It should be noted, however, that strict criteria for FNAC are applied in our centers, reducing the number of examinations and, especially, we only included ^18^FDG-avid nodules and postoperative histological diagnosis was mandatory for inclusion.

Fourth, some studies suggest that the AUS/FLUS category should be further subdivided into AUS with cytologic atypia (higher risk for malignancy) and FLUS with architectural atypia (lower risk for malignancy) [23]. However, this approach has not yet been widely adopted in clinical practice and we cannot evaluate the potential impact of subdividing AUS and FLUS classes in our patients.

Fifth, we did not compare PET/CT data with ultrasound (US) and molecular biomarkers.

Finally, even if the prevalence was in line with current rates reported in the literature, the enrolled population is relative small and the validation of the current model in a prospective study including a larger number of cases is warranted to confirm these results.

Ultrasound is one of the principal steps in the initial workup of thyroid nodules and different risk stratification systems are now recommend FNAC dependent on nodule size and various combinations of US characteristics with an incremental risk of malignancy [22]. However, US remains an operator-dependent procedure and a reliable comparison of US results was precluded in our study as US examinations were performed by different sonographers in different centres. In addition, despite some authors support the use of ultrasound to risk stratify nodules with indeterminate cytology no clear recommendations are provided regarding (re)interpretation of US characteristics after FNAC has resulted in indeterminate cytology [24, 25]. Notably, currently available US risk stratification systems have been evaluated against papillary carcinoma while follicular-type malignancies typically have a different US appearance and caution was advised when using US to evaluate follicular-patterned lesion and capture FTCs [26].

Three different approaches characterize thyroid molecular tests [27]. One aims to exclude (rule-out) and the other aims to confirm (rule-in) malignancy in the indeterminate category. The rule-out test is offered by the Veracyte company and consists in the evaluation of several mRNA to optimize NPV and is called Afirma GEC. The rule-in test is developed by the University of Pittsburg and commercialized by CBLPath and known as ThyroSeq. It is based on next generation sequencing (NGS) for point mutations and gene fusions in known thyroid cancer related genes. Another test available and belonging to the rule-in tests is the ThyGenX-ThyraMIR commercialized by Interpace Diagnostics. Such tests are currently not available in Europe, even in referral centers, due to the high costs.

A different approach, available in many referral centers also in Europe, is based on gene mutations analysis mutational panel (BRAF, H-N-K-RAS, RET/PTC PAX8/PPAR-gamma). On the other hand, BRAF mutation analysis is 100% predictive of papillary thyroid carcinoma (high PPV), but most cancers are BRAF-negative (very low NPV); mutations of RAS-family genes mutations are also observed in follicular adenomas and the prevalence of *PAX8/PPARγ* rearrangements is generally limited in IC nodules with no cases reported in some studies.

Finally, all methods largely depend on local cancer prevalence and pretest probabilities, and no definitive guidelines exist, consequently a locally adapted multimodality stepwise approach, ideally combining one rule-in and one rule-out test, likely offers the most accurate diagnosis [2]. Accordingly, the potential improvement generated by the integration of PET/CT radiomics, molecular and/or other imaging biomarkers certainly deserves to be further explored, and validation of the current model in a prospective study including a larger number of cases is warranted to confirm the present results.

We demonstrated that the combination of PET/CT-based radiomic signature and Bethesda classes (i.e., III vs IV) in a predictive model increases the accuracy of risk stratification compared to Bethesda system and PET/CT alone. This combined approach may reduce the number of diagnostic lobectomies, with associated advantages in terms of costs and quality of life for patients.

## Supplementary Information

Supplementary Information

## Data Availability

The data presented in this study are available in the article or supplementary files.

## Code availability

The codes used during the study are available from the corresponding author on reasonable request.
