# Supplementary material for: Radiomics analysis improves 18FDG PET/CT-based risk stratification of cytologically indeterminate thyroid nodules
Source: Endocrine. 2021 Sep 1;75(1):202–10. doi: 10.1007/s12020-021-02856-1 (PMC8763930; doi:10.1007/s12020-021-02856-1)

**Supplementary Information**

**Radiomics analysis improves ^18^FDG PET/CT-based risk stratification of cytologically indeterminate thyroid nodules**

**Endocrine**

Luca Giovanella^1,2^*, Lisa Milan^1^, Arnoldo Piccardo^3^, Gianluca Bottoni^3^, Marco Cuzzocrea^1^, Gaetano Paone^1^ and Luca Ceriani^1,4^

^1^ Clinic for Nuclear Medicine and Molecular Imaging, Imaging Institute of Southern Switzerland, Ente Ospedaliero Cantonale, 6500 Bellinzona, Switzerland

^2^ Clinic for Nuclear Medicine, University Hospital and University of Zurich, Zurich, Switzerland;

^3^ Department of Nuclear Medicine, E.O. “Ospedali Galliera”, Genoa, Italy;

^4^ Faculty of Biomedical Sciences, Università della Svizzera Italiana (USI), Lugano, Switzerland;

*Corresponding author: Luca Giovanella, [luca.giovanella@eoc.ch](mailto:luca.giovanella@eoc.ch)

**Extraction of radiomics features**

For the extraction of radiomics features from PET/CT imaging, we used the PyRadiomics platform, a software package compliant with the Image Biomarker Standardization Initiative (IBSI). PyRadiomics is a flexible, open-source platform implemented in the Python programming language and capable of extracting a large panel of engineered features from medical images. These features are subdivided into the following classes:

- First Order Statistics (19 features)
- Shape-based (3D) (16 features)
- Shape-based (2D) (10 features)
- Gray Level Cooccurence Matrix (GLCM, 24 features)
- Gray Level Run Length Matrix (GLRLM, 16 features)
- Gray Level Size Zone Matrix (GLSZM, 16 features)
- Neighbouring Gray Tone Difference Matrix (NGTDM, 5 features)
- Gray Level Dependence Matrix (GLDM, 14 features)

The shape-based features measure the dimensions and depict the geometric properties of the lesion. The first-order statistics features describe the presence of areas with statistically different metabolic activity without taking into account their distribution within the lesion, while the matrix-based features analyse spatial distribution of these areas and their mutual relationship. In the last group, the analysis is performed by applying multiple mathematical models to the data organised in the form of matrices. Supplementary Table 1 summarises the default parameters we used to extract radiomics features. Supplementary Table 2 enumerates the extracted features; those included in the LASSO logistic regression are in bold type. Their mathematical description is available in the PyRadiomics online documentation [https://pyradiomics.readthedocs.io/en/latest/features.html].

**Supplementary Table 1** Parameters used to extract radiomics features

| **Calculation setting** | **Default** |
| --- | --- |
| Approach | 3D |
| Interpolation   - Voxel dimension (mm) - Interpolation method - ROI interpolation method | Yes   - 3x3x3 - B-Spline - Nearest-Neighbor |
| Re-segmentation   - Range (SUV) - Outliers | - No - No |
| Discretization | Fixed bin size: 64 bins |

**Supplementary Table 2** List of extracted radiomics features

| **First-order features** | | | | |
| --- | --- | --- | --- | --- |
| *Histogram-based features* | | | | |
| **10Percentile***  90Percentile  Energy  **Entropy***  InterquartileRange  **Kurtosis***  **Maximum***  MeanAbsoluteDeviation  Mean  Median  **Minimum***  Range  RobustMeanAbsoluteDeviation  RootMeanSquared  **Skewness***  TotalEnergy  **Uniformity***  Variance | | | | |
| **Shape-based features** | | | | |
| *3D shape features* | | | | |
| **Elongation***  **Flatness***  LeastAxisLength  MajorAxisLength  Maximum2DDiameterColumn  Maximum2DDiameterRow  Maximum2DDiameterSlice  Maximum3DDiameter  MeshVolume  MinorAxisLength  **Sphericity***  SurfaceArea  SurfaceVolumeRatio  **VoxelVolume*** | | | | |
| **Second-order (texture) features** | | | | |
| *GLCM* | *GLDM* | *GLRLM* | *GLSZM* | *NGTDM* |
| **Autocorrelation***  **ClusterProminence***  **ClusterShade***  **ClusterTendency***  Contrast  Correlation  DifferenceAverage  **DifferenceEntropy***  DifferenceVariance  Id  Idm  Idmn  Idn  Imc1  Imc2  InverseVariance  JointAverage  JointEnergy  JointEntropy  **MCC***  **MaximumProbability***  SumAverage  SumEntropy  **SumSquares*** | DependenceEntropy  DependenceNonUniformity  DependenceNonUniformityNormalized  DependenceVariance  GrayLevelNonUniformity  **GrayLevelVariance***  **HighGrayLevelEmphasis***  LargeDependenceEmphasis  **LargeDependenceHighGrayLevelEmphasis***  **LargeDependenceLowGrayLevelEmphasis***  **LowGrayLevelEmphasis***  SmallDependenceEmphasis  SmallDependenceHighGrayLevelEmphasis  **SmallDependenceLowGrayLevelEmphasis*** | GrayLevelNonUniformity  **GrayLevelNonUniformityNormalized***  **GrayLevelVariance***  **HighGrayLevelRunEmphasis***  LongRunEmphasis  **LongRunHighGrayLevelEmphasis***  **LongRunLowGrayLevelEmphasis***  **LowGrayLevelRunEmphasis***  **RunEntropy***  RunLengthNonUniformity  RunLengthNonUniformityNormalized  RunPercentage  RunVariance  ShortRunEmphasis  **ShortRunHighGrayLevelEmphasis***  **ShortRunLowGrayLevelEmphasis*** | GrayLevelNonUniformity  **GrayLevelNonUniformityNormalized***  **GrayLevelVariance***  **HighGrayLevelZoneEmphasis***  LargeAreaEmphasis  LargeAreaHighGrayLevelEmphasis  LargeAreaLowGrayLevelEmphasis  **LowGrayLevelZoneEmphasis***  SizeZoneNonUniformity  SizeZoneNonUniformityNormalized  SmallAreaEmphasis  **SmallAreaHighGrayLevelEmphasis***  **SmallAreaLowGrayLevelEmphasis***  ZoneEntropy  ZonePercentage  ZoneVariance | Busyness  Coarseness  **Complexity***  Contrast  Strength |

*****Bold type indicates RFs uncorrelated with tumour volume (MTV) and its uptake (SUV_max_), which were included in the LASSO logistic regression; they comprise 7 first-order features, 4 3D shape features, and 30 second-order matrix features.

**LASSO logistic regression**

The determination of the optimised regularisation parameter λ, which ensured that the model had the least deviance improving both the prediction accuracy and the interpretability, was selected via the minimum criteria (i.e. the value of λ that provides the minimum mean cross-validated error). The optimal tuning of this parameter allows for obtaining non-zero coefficients only for the more predictive features. The optimal values of the penalty parameter λ were determined by 10-fold cross-validations.

**Supplementary Fig. 1** Feature selection using the LASSO logistic regression with 10-fold cross-validation. (A) In the LASSO logistic model, the minimum standard is adopted to obtain the value of the parameter λ by 10-fold cross-validation. The λ value was confirmed as 0.089. (B) Coefficient sectional view plotted against the log (λ) magnitude. Based on 10-fold cross-validation, the optimal λ corresponding to two non-zero coefficients (indicated by a vertical line in the plot) were selected

**A.**

**B.**
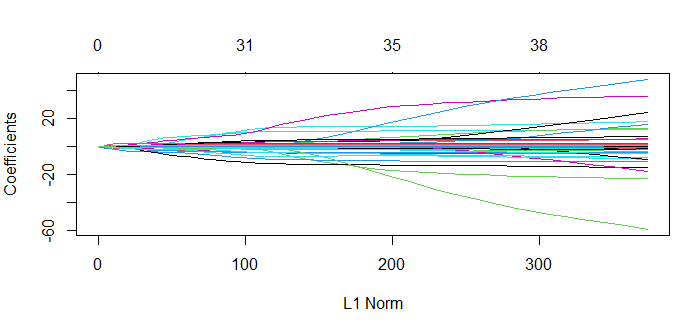

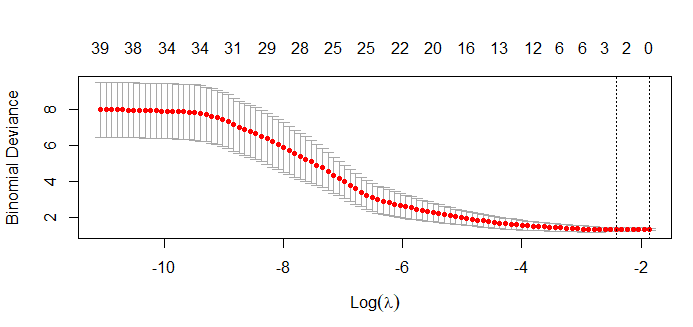

Supplement: Supplementary file 1 — Supplementary Information [file 12020_2021_2856_MOESM1_ESM.docx]
